# Hyperosmotic stimulus induces reversible angiogenesis within the hypothalamic magnocellular nuclei of the adult rat: a potential role for neuronal vascular endothelial growth factor

**DOI:** 10.1186/1471-2202-6-20

**Published:** 2005-03-24

**Authors:** Gérard Alonso, Evelyne Galibert, Anne Duvoid-Guillou, Anne Vincent

**Affiliations:** 1 CNRS UMR 5203; INSERM U661; Univ. Montpellier I and II; Institut de Génomique Fonctionnelle, Departement d'Endocrinologie, 141 Rue de la Cardonille, Montpellier F-34094 Cedex 5, France

## Abstract

**Background:**

In mammals, the CNS vasculature is established during the postnatal period via active angiogenesis, providing different brain regions with capillary networks of various densities that locally supply adapted metabolic support to neurons. Thereafter this vasculature remains essentially quiescent excepted for specific pathologies. In the adult rat hypothalamus, a particularly dense network of capillary vessels is associated with the supraoptic (SON) and paraventricular (PVN) nuclei containing the magnocellular neurons secreting vasopressin and oxytocin, two neurohormones involved in the control of the body fluid homoeostasis. In the seventies, it was reported that proliferation of astrocytes and endothelial cells occurs within these hypothalamic nuclei when strong metabolic activation of the vasopressinergic and oxytocinergic neurons was induced by prolonged hyperosmotic stimulation. The aim of the present study was to determine whether such proliferative response to osmotic stimulus is related to local angiogenesis and to elucidate the cellular and molecular mechanisms involved.

**Results:**

Our results provide evidence that cell proliferation occurring within the SON of osmotically stimulated adult rats corresponds to local angiogenesis. We show that 1) a large majority of the SON proliferative cells is associated with capillary vessels, 2) this proliferative response correlates with a progressive increase in density of the capillary network within the nucleus, and 3) SON capillary vessels exhibit an increased expression of nestin and vimentin, two markers of newly formed vessels. Contrasting with most adult CNS neurons, hypothalamic magnocellular neurons were found to express vascular endothelial growth factor (VEGF), a potent angiogenic factor whose production was increased by osmotic stimulus. When VEGF was inhibited by dexamethasone treatment or by the local application of a blocking antibody, the angiogenic response was strongly inhibited within the hypothalamic magnocellular nuclei of hyperosmotically stimulated rats.

**Conclusion:**

This study shows that the functional stimulation of hypothalamic magnocellular neurons of adult rats induces reversible angiogenesis via the local secretion of neuronal VEGF. Since many diseases are driven by unregulated angiogenesis, the hypothalamic magnocellular nuclei should provide an interesting model to study the cellular and molecular mechanisms involved in the regulation of angiogenesis processes within the adult CNS.

## Background

Within the CNS, capillary blood vessels form a network of highly interconnected tubes that direct and maintain blood flow throughout the different regions. In the adult CNS, the vascular supply is not homogenous and marked differences exist in the capillary density present within specific brain regions. Since blood glucose represents the major metabolic support of neurons, it has been proposed that the density of the vasculature network is related to the different levels of the metabolic activity [1]. It is generally admitted that the adult vasculature is essentially quiescent and that adjustment of blood supply to increased metabolic activity occurs locally via modifications of the diameter of blood vessels [2]. However previous studies have suggested that chronic stimulation of specific neuronal systems was able to locally modify the blood supply via angiogenesis. For instance, rearing rats in a complex environment was found to increase the capillary density within the visual cortex [3], whereas prolonged motor activity was reported to induce angiogenesis within the cerebellar cortex [4] and primary motor cortex [5]. The magnocellular nuclei of the hypothalamus have long been shown to contain a particularly high density of capillaries [6-8]. These hypothalamic nuclei contain two populations of magnocellular neurons that synthesize two peptidic neurohormones, vasopressin (VP) and oxytocin (OT) that play major roles in the control of body fluid balance. Since these magnocellular neurons synthesize huge amounts of VP and OT throughout life span, it has been admitted that hypervascularization of these nuclei facilitates the supply of circulating glucose needed for sustaining a high metabolic activity [9]. Moreover, the activity of hypothalamic magnocellular neurons is directly regulated by changes in plasma osmotic pressure and their metabolic activity can be chronically stimulated by prolonged osmotic stimuli [10]. Interestingly, it has been reported that proliferation of glial and endothelial cells could be observed within the hypothalamic magnocellular nuclei in animals submitted to prolonged osmotic stimulus [11]. In this context, the aim of our study was to determine whether prolonged metabolic activation of magnocellular neurons was able to modify the vasculature throughout the hypothalamic nuclei via local angiogenesis. Our results show that hyperosmotic stimuli induce local proliferation of SON capillary endothelial cells, leading to a reversible increase in the density of the capillary network within the nuclei. We also show that contrasting with most CNS neurons, magnocellular hypothalamic neurons continue to express high levels of VEGF throughout adulthood and that this endogenous cytokine is at least in part responsible for the local angiogenesis induced by hyperosmotic stimuli.

## Results

In the present study, the observations were restricted to brain sections passing through the hypothalamus that contained the different magnocellular nuclei, including the supraoptic (SON), paraventricular (PVN), and accessory (AN) nuclei. As compared with the other hypothalamic magnocellular nuclei, the SON essentially contains VP and OT neurons and has a clearly defined anatomical localization (along the lateral border of the optic chiasma). For reasons of clarity, the data reported here will therefore mostly concern the SON, although the present findings apply for all the hypothalamic magnocellular nuclei.

### Hyperosmotic stimulus induces reversible angiogenesis within the SON

#### The kinetics of cell proliferation

**Figure 1:**
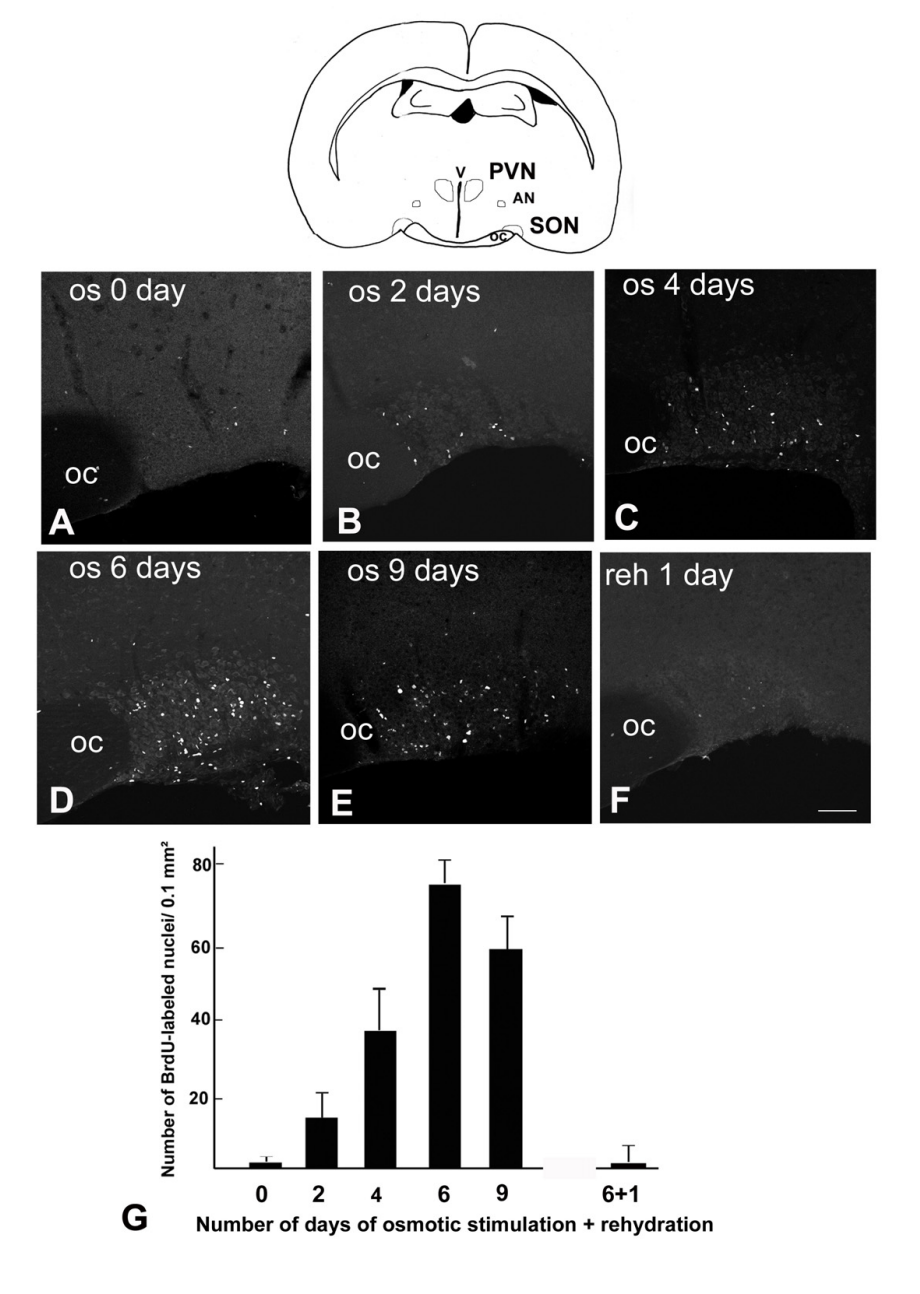
**Hyperosmotic stimuli induce cell proliferation within the SON**. BrdU was administrated to rats drinking 2% saline during various increasing periods, or to rats rehydrated after 6 days of osmotic stimulation, and the animals were fixed 5 hours after the BrdU administration. **A-F**: Stack confocal images (10 μm-thick) of sections labeled for BrdU. **G**: Quantitative evaluation of the number of BrdU-labeled nuclei detected within the SON. Cell proliferation is virtually absent in control rats (0 day of stimulation), progressively increases from 2 to 6 days of osmotic stimulation, and is abolished after 1 day of rehydration in 6 days stimulated rats. Data represent means ± SEM of counts made on at least 4 SON areas per rats in 3 rats per experiment. The schematic drawing illustrates the anatomical locations of the SON and other hypothalamic magnocellular nuclei. AN: accessory nucleus; OC: optic chiasma; PVN: paraventricular nucleus; SON: supraoptic nucleus. Scale bar: 100 μm.

To determine the kinetics of the proliferative response to hyperosmotic stimulus adult rats were provided with 2 % saline drinking water during 0 (controls), 1, 2, 4, 6 and 9 days. At the end of the stimulation, animals were injected with BrdU and fixed 5 hours later. In control rats, BrdU-labeled nuclei were never detected within the SON (Fig. 1A), although they were constantly detected within the germinative zones including the dentate gyrus of the hippocampus [12] and the subventricular zone of the lateral ventricle [13] (not shown). In all the stimulated rats by contrast, BrdU-labeled nuclei were detected within both the germinative zones and the SON (Fig. 1B–E) and other magnocellular hypothalamic nuclei including the PVN and accessory nuclei (not shown). The quantification of BrdU-labeled nuclei in the SON indicated that this proliferative response was maximum after to 6 days of osmotic stimulation, and then slightly decreased (Fig. 1G). The same protocol was used to study the proliferative response within the SON of 6 days osmotically stimulated rats that were rehydrated for 1, 2 and 3 days: in all these animals, only scarce BrdU-labeled nuclei could be detected within the SON after 1 day rehydration (Fig. 1F and 1G), indicating that cell proliferation was rapidly stopped at the end of the stimulus.

#### The nature of proliferating cells

In this part of the study, BrdU was injected to stimulated rats provided with 2% saline drinking water during 6 days and the animals were fixed 5 hours later. In order to identify the nature of the cells that proliferate within the SON, we looked for the possible association of BrdU-labeled nuclei with the main cell types contained in the hypothalamic nucleus, including the neurons, glial cells and capillary endothelial cells.

##### Neurons

The SON essentially contains magnocellular neurons that were labeled by specific antibodies against VP or OT. A mixing of both antibodies was used to label the whole population of SON neurons. In all sections double immunostained for VP + OT and BrdU, BrdU-labeled nuclei never appeared associated with VP- or OT-labeled neuronal cell bodies (not shown).

##### Glial cells

**Figure 2:**
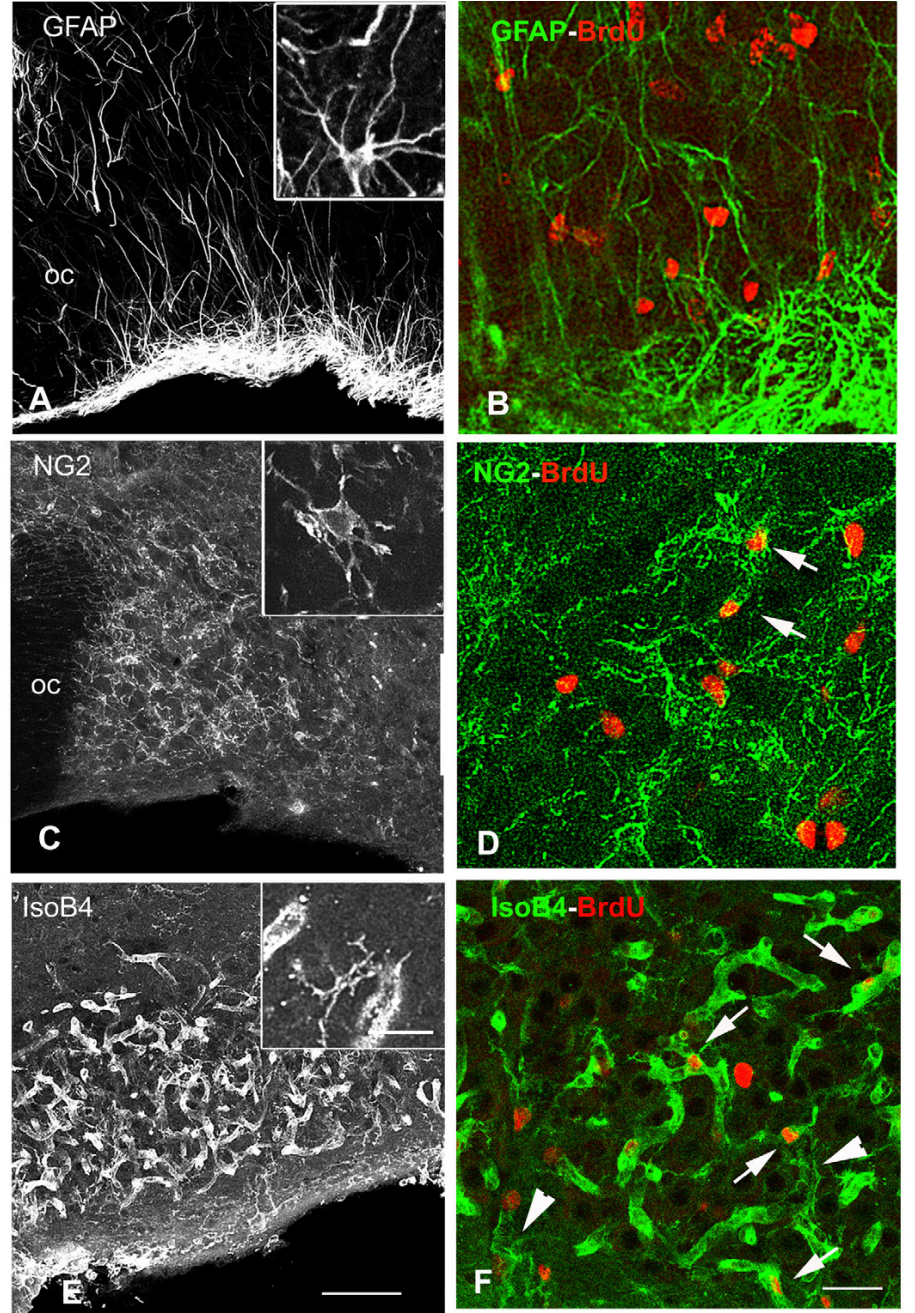
**Cell proliferation concerns a minority of SON glial cells. **BrdU was administrated to osmotically stimulated rats drinking 2% saline during 6 days and the animals were fixed 5 hours after the BrdU administration. **A, C, E**: Stack confocal images (10 μm-thick) of sections labeled for different glial markers. **A**: GFAP immunostaining is associated with the cell bodies of SON astrocytes localized to the ventral border of the nucleus and with their elongated processes projecting dorsally throughout the core of the nucleus. **C**: NG2 immunostaining is associated with small ramified cells dispersed throughout the nucleus. **E**: IsoB4 labeling is associated both with typical ramified microglial cells dispersed within the nucleus and with some tubular, vessel-like structures. Insets show high magnification of the different labeled cell types. **B, D, and F**: Merged confocal images of sections double labeled for BrdU and for one the different glial markers. Within the SON, BrdU-labeled nuclei are never associated with GFAP-labeled astrocytes (**B**) and IsoB4-labeled isolated microglial cells (arrow heads in **F**), whereas they are associated with some NG2-labeled cells (arrows in **D**) and with numerous vessel-like structures labeled with the lectin IsoB4 (arrows in **F**). GFAP: glial fibrillary acidic protein; IsoB4: isolectin B4; NG2: NG2 proteoglycan; OC: optic chiasma. Scale bars: A, C, E = 100 μm; B, D, F = 25 μm; Insets = 25 μm.

**Figure 3:**
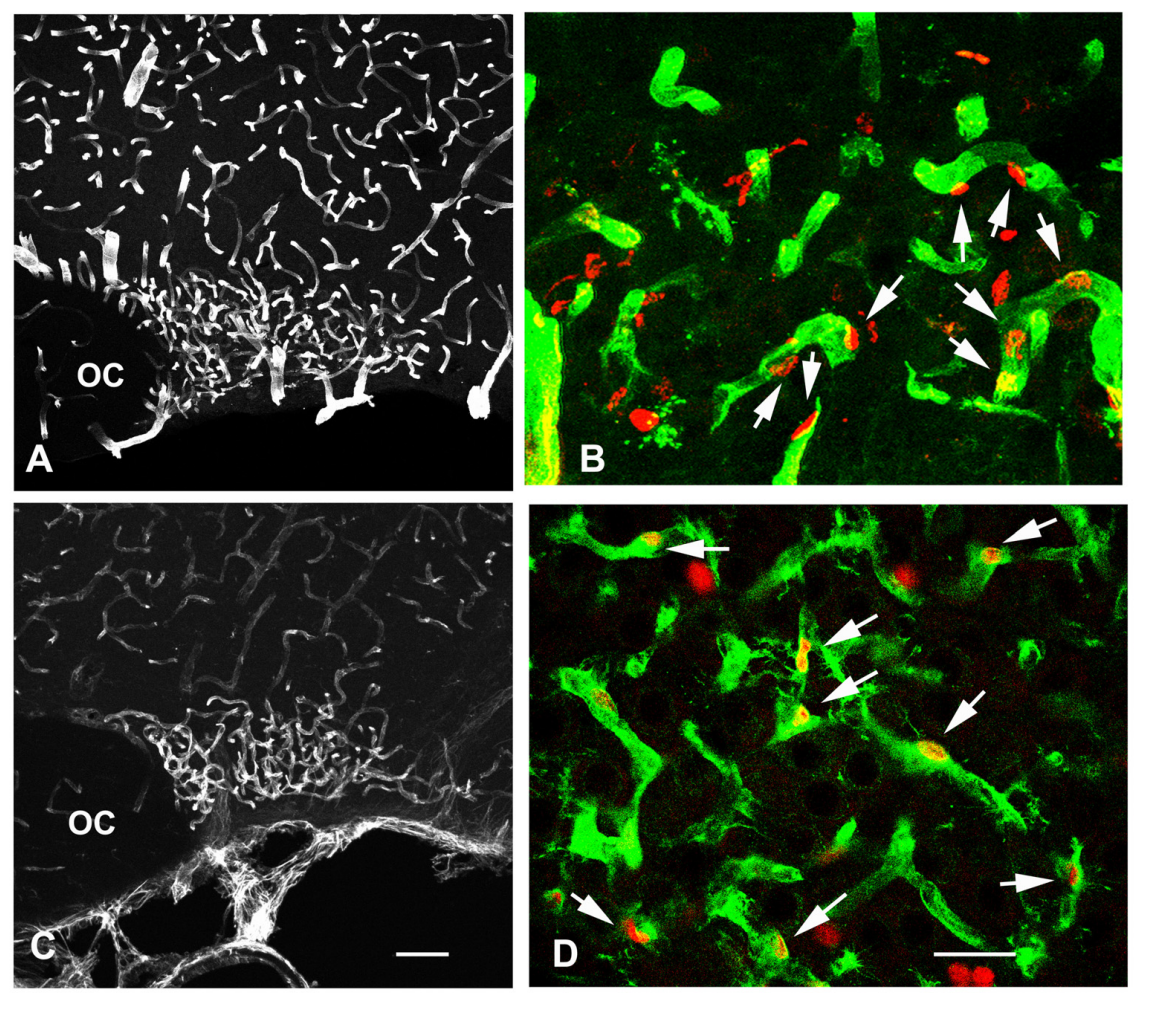
**Cell proliferation mostly concerns SON capillary endothelial cells**. BrdU was administrated to osmotically stimulated rats drinking 2% saline during 6 days, and the animals were fixed 5 hours after the BrdU administration. **A, C**: Stack confocal images (10 μm-thick) of sections labeled for endothelial cell markers. EBA immunostaining is associated with vessels located within the SON and the surrounding regions (**A**), whereas nestin immunostaining is associated with SON vessels, but only faintly labels those vessels located in the surrounding regions (**C**). **B, D**: Merged confocal images of sections double labeled for BrdU and for one of the two the endothelial cell markers. Throughout the SON, BrdU-labeled nuclei are frequently associated with vessel structures labeled for EBA (arrows in **B**) or nestin (arrows in **D**). EBA: endothelial brain antigen; OC: optic chiasma. Scale bars: A, C = 100 μm; B, D = 25 μm.

The SON contains three main glial cell types including 1) astrocytes that were labeled by the means of an antibody against GFAP, an intermediate filament protein specifically expressed by this cell type [14], 2) oligodendrocyte precursor cells that were labeled by an antibody against the proteoglycan NG2 [15,16]), and 3) microglial cells that were labeled by the isolectin B4 from *Griffonia simplicifolia *(IsoB4, recognizing glycoproteins that are specifically associated with the limiting membrane of resting and activated microglia [17], and with capillary vessels endothelial cells[18]). These different glial cells markers produced typical labeling patterns within the SON: 1) GFAP-labeling was associated with astrocytic cell bodies localized to the ventral surface of the nucleus and with their elongated processes extending dorsally throughout the core of the nucleus (Fig. 2A), 2) NG2-labeling was associated with small cells exhibiting numerous radiating processes that were homogeneously dispersed throughout the SON and the surrounding regions (Fig. 2C), and 3) IsoB4 labeling was associated with both typical ramified microglial cells dispersed throughout the nucleus and with a number of capillary vessels (Fig. 2E). In double labeled sections, BrdU-labeled were rarely associated with the GFAP-labeled astrocytic cell bodies located in the ventral portions of the nucleus (Fig. 2B) and with the IsoB4-labeled isolated microglial cells dispersed throughout the nucleus (Fig. 2D). On the other hand, labeled nuclei were frequently associated with IsoB4-labeled capillary vessels (Fig. 2D) and to a lesser extent with NG2-labeled cells bodies dispersed throughout the SON (Fig. 2F).

##### Capillary endothelial cells

In addition to the isolectin B4 (see above), the endothelial cells composing the SON capillary vessels were labeled with specific antibodies against either nestin (an intermediate filament protein that is expressed by these cells during prenatal and early postnatal periods [19,20] or the endothelial barrier antigen (EBA, a protein that is expressed by endothelial cells in the mature rat brain) [21]. In all the adult rats examined (2–3-month-old), the phenotypic expression of SON capillary vessels markedly differed from those located in the surrounding brain regions: SON capillaries always exhibited moderate to intense immunoreactivity to both EBA and nestin, whereas all the vessels located in the surrounding regions were EBA-positive but nestin-negative (Fig. 3A and 3C). The examination of double labeled sections indicated that, a large majority of the BrdU-labeled nuclei detected throughout the SON was associated with capillary-like processes labeled for EBA (Fig. 3B) or nestin (Fig. 3D). These data indicate that the cell proliferation occurring within the SON following osmotic stimuli preferentially concern the capillary endothelial cells.

### Hyperosmotic stimulus reversibly modifies the organization of SON vessels

Since proliferation of endothelial cells generally results in the formation of new vessels, we then examined the consequences of prolonged osmotic stimuli on the SON vascularization. To do this, we compared the anatomical organization and the phenotypic expression of SON capillaries in normally hydrated rats (controls), and in stimulated rats drinking 2% saline for various periods (1 to 9 days).

#### Anatomical organization of SON vessels

**Figure 4:**
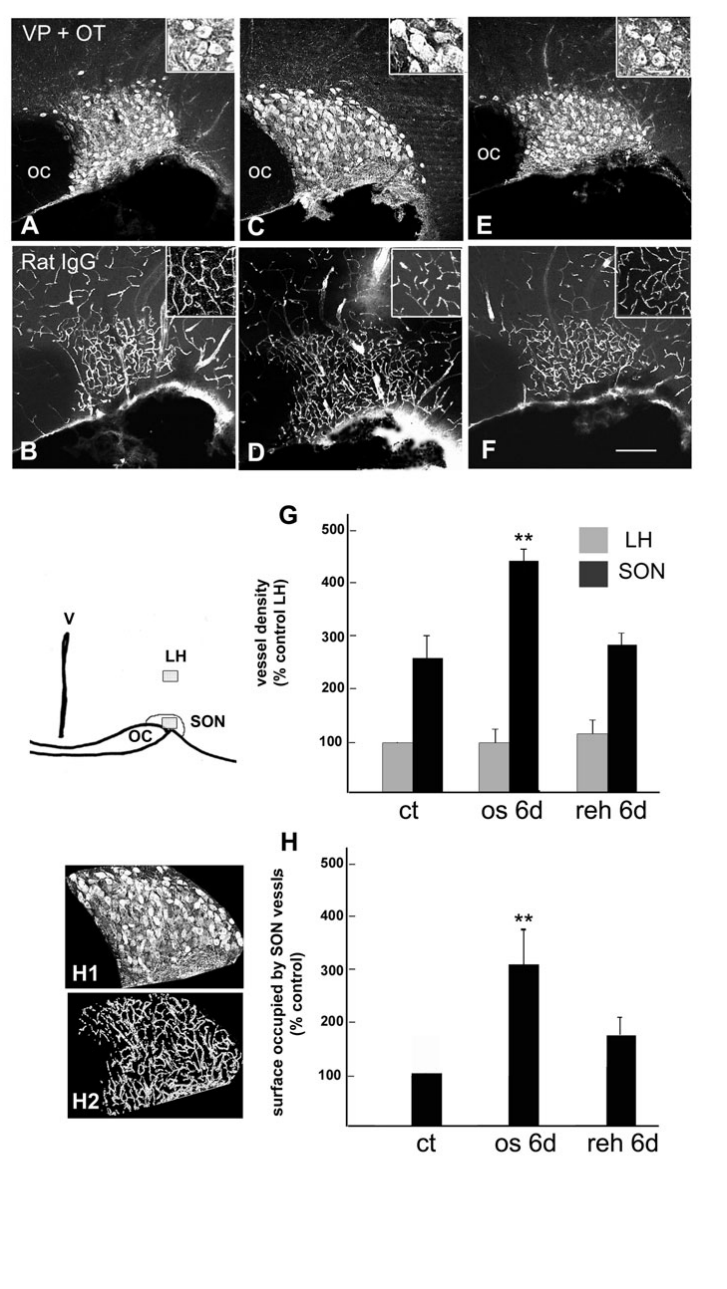
**Hyperosmotic stimulus reversibly modifies the anatomical organization of SON vessels**. Freshly dissected brains of rats normally hydrated (**A-B**); osmotically stimulated during 6 days (**C-D**) and rehydrated during 6 days following 6 days of osmotic stimulation (**E-F**) were fixed by immersion in the fixative and the content of blood vessels was labeled by an antibody against Rat IgG. **A-F**: Stack confocal images (10 μm-thicks) of sections double immunostained for Rat IgG and VP + OT. Insets show the morphology of VP or OT SON neurons (**A, C, E**), and the organization of Rat IgG-labeled vessels in the lateral hypothalamus (**B, D, F**). **G**: Quantitative evaluation of the vessels density within the SON and the lateral hypothalamus. For each section analyzed, a 100 point grid was centered on the core of the nucleus or on the overlying lateral hypothalamus (as shown in the schematic drawing), and the vessel density was estimated by scoring vessel contacts with the bars of the grid. The data are expressed as percent of the vessel density determined in the lateral hypothalamus overlying the SON of control rats. **H**: Quantitative evaluation of the total amount of SON vessels. For each section analyzed the SON surface was first determined by delineating the area containing the neuronal cell bodies labeled for OT or VP (H1 shows the SON area selected from the image C). The surface occupied by vessels was then quantified on binary images of the Rat-IgG-labeled structures within this SON area (H2 shows the binary image of labeled vessels selected from the image D). All the quantifications were performed on at least 4 sections containing the middle portions of the SON (i.e. the largest SON areas), for at least 3 rats per experiment. Note that the size of individual VP or OT immunostained neurons is highly increased in the SON of stimulated rat (inset C) as compared with control (inset A) or rehydrated rat (inset E). Double asterisk: P < 0.01, Mann-Whitney test, statistically different from the control SON. ct: control, normally hydrated; IgG: type G immunoglobulin; LH: lateral hypothalamus; OC: optic chiasma; OT: oxytocin; os 6d: osmotically stimulated during 6 days; reh 6d: rehydrated during 6 days following 6 days stimulation; SON: supraoptic nucleus; VP: vasopressin. Scale bars: A-C = 100 μm. Insets A, C, D = 25 μm.

In this part of the study, the rats were decapitated and brains were incubated in a 4% paraformaldehyde solution in order to fix the serum proteins contained within blood vessels. The organization of capillary vessels was then visualized on 50-μm-thick vibratome sections, by labeling the intra-vascular content by an antibody against Rat IgG conjugated to Alexa-fluor 488. The examination of anti-rat-IgG-labeled sections indicated that 1) in control, normally hydrated adult rats, the density of capillary vessels was significantly higher within the SON area (identified as the area containing the cell bodies of VP and OT neurons) than in the surrounding brain regions (Fig. 4A), and 2) in 6 days osmotically stimulated rats, this density was significantly increased within the SON area but not in the surrounding regions (Fig. 4C–D). Moreover, during the osmotic stimulation, the size of the individual VP and OT neurons was progressively increased, leading to an extension of the SON area (Fig. 4A–C). A quantitative analysis of the Rat-IgG-labeled sections indicated that after 6 days of stimulation, the surface occupied by SON capillaries was dramatically increased (× 3 to 4) as compared with the SON of normally hydrated rat. These modifications were reversed by the rehydration of stimulated animals: the density of SON capillaries progressively decreased when 6 days stimulated rats were rehydrated, and was reestablished to control levels in animals rehydrated for 6 days (Fig. 4G and 4H).

#### Phenotypic expression of SON vessels

**Figure 5:**
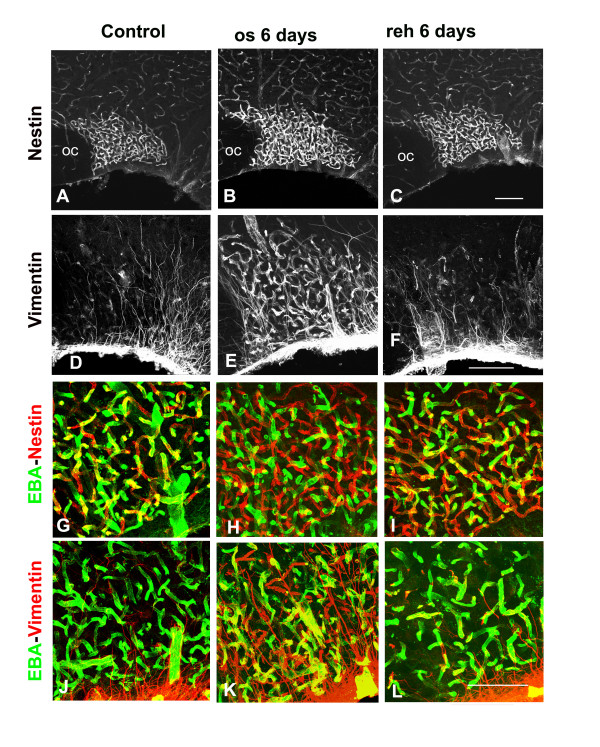
**Hyperosmotic stimulus reversibly increases the expression of nestin and vimentin by SON capillary vessels**. **A-F**: Stack confocal images (10 μm-thick) of sections immunostained for nestin (**A-C**) or vimentin (**D-F**) in rats normally hydrated (**A **and **D**), osmotically stimulated during 6 days (**B **and **E**), and rehydrated during 6 days following 6 days of osmotic stimulation (**C **and **E**). In all the rats, nestin immunostaining of vessels is more intense in the SON than in the surrounding regions, whereas the number and staining intensity of immunostained SON vessels increases in the osmotically stimulated rats (**B **vs **A**), and return to control levels in the rehydrated rats (**C**). In all the rats, intense vimentin immunostaining is associated with the cell body of astrocytes located along the SON ventral border, and with their elongated processes projecting throughout the nucleus (**D-F**), whereas vimentin-immunostained vessels are only detected in the SON of the stimulated rat (**E**). **G-L**: Merged confocal images of sections double immunostained for EBA and either nestin (**G-I**) or vimentin (**J-L**) in rats normally hydrated (**G **and **J**), osmotically stimulated during 6 days (**H **and **K**), and rehydrated during 6 days following 6 days of osmotic stimulation (**I **and **L**). In control (**G **and **I**) and rehydrated (**I **and **L**) rats, numerous SON vessels are double-immunostained for EBA and nestin (yellow vessels in **G **and **I**), whereas in the stimulated rat, number of capillary vessels labeled for nestin appear EBA-negative (red vessels in **H**). As compared with control (**J**) and rehydrated (**L**) rats, the SON of the osmotically stimulated rat contain numerous vessels double immunostained for EBA and vimentin or (yellow vessels in **K**), or vimentin-positive but EBA-negative (red vessels in **K**). Control: normally hydrated rat; EBA: endothelial brain antigen; OC: optic chiasma; os 6 days: rat osmotically stimulated during 6 days; reh 6 days: rat rehydrated during 6 days following 6 days of osmotic stimulation. Scale bars: A-C = 100 μm; D-F = 100 μm; G-L= 50 μm.

In a second step we looked for the expression by SON vessels of nestin or vimentin, two intermediate filament proteins that are expressed by the endothelial cells constituting newly formed capillary vessels in the developing CNS or in brain tumors [19,20,22,23]. In control adult rats, the large majority of the capillary vessels present throughout the brain exhibited intense EBA immunostaining, but faint if any immunostaining for nestin or vimentin. By contrast, SON capillary vessels always exhibited moderate to intense immunostaining for EBA and nestin, whereas vimentin immunostaining was essentially associated with SON astrocytic cell bodies and processes [24] (Fig. 5A and 5D). In osmotically stimulated rats, the SON was found to contain an increased amount of nestin-immunostained vessels, frequently exhibiting higher staining intensity than in the control rats (Fig. 5B). Contrasting with control rats, vimentin immunostaining was now associated with both SON astrocytes and capillary vessels, whereas vessels located in the surrounding brain regions remained vimentin-negative (Fig. 5E). As observed for their anatomical organization, rehydration of 6 days osmotically stimulated rats induced progressive modifications of the phenotype of SON vessels, and their expression of nestin and vimentin was reestablished to control levels after 6 days of rehydration (Fig. 5C and 5F). The examination of sections double immunostained for EBA and either nestin or vimentin further indicated that, as compared with control or rehydrated rats, the SON of osmotically stimulated rats contained an increased amounts of capillary processes that were immunostained for either nestin or vimentin but appeared EBA negative (Fig. 5 G–L).

### SON angiogenesis is related to neuronal vascular endothelial growth factor

#### SON neurons maintain the expression of VEGF throughout adulthood

**Figure 6:**
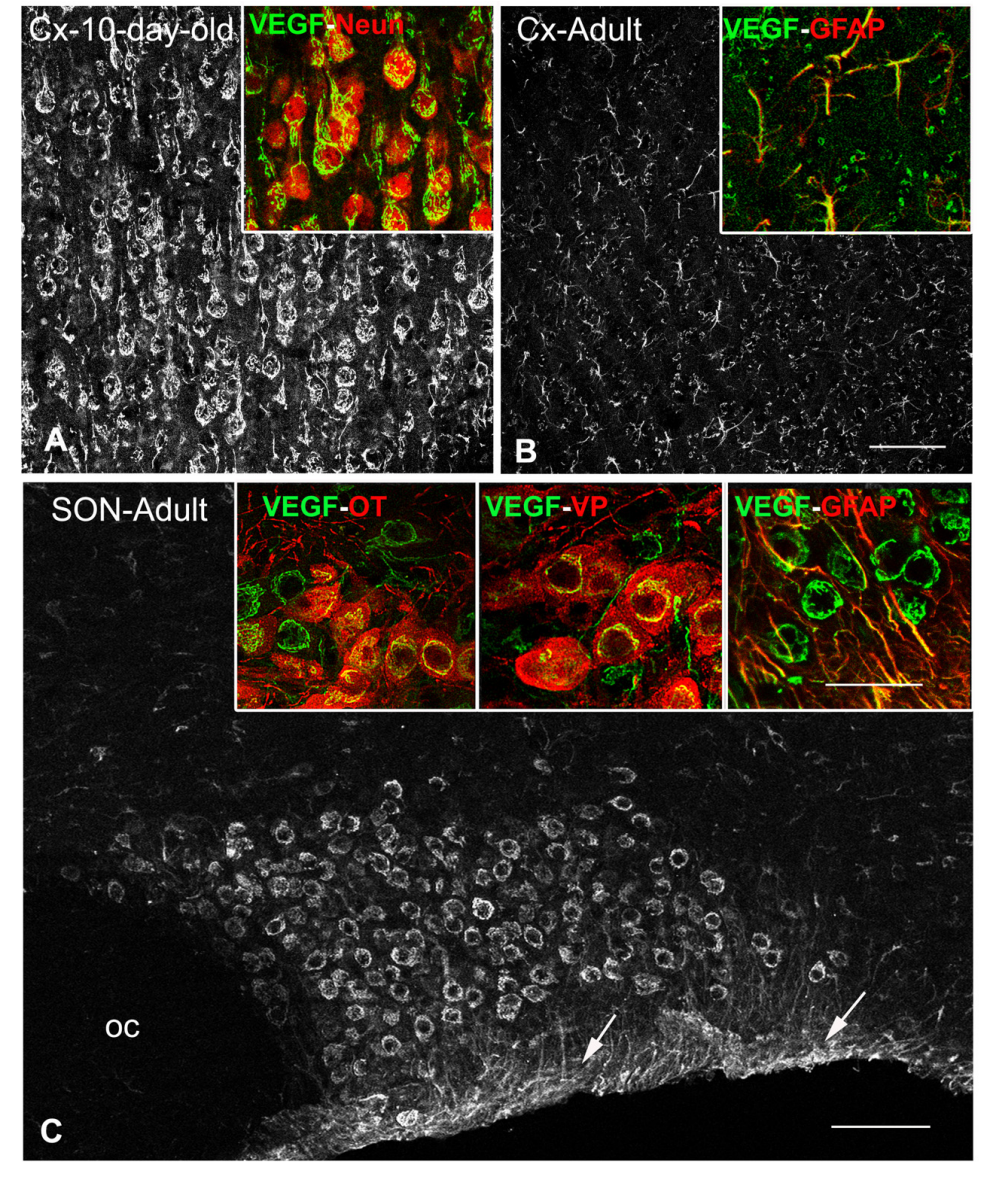
**VEGF is expressed by adult SON neurons**. **A-C**: Stack confocal images (10 μm-thick) of sections immunostained for VEGF. In the cortex of newborn (**A**, 10-day-old) and adult (**B**, 3-month-old) rats, VEGF immunostaining is associated with neuron-like and glial-like structures, respectively. Insets: merged confocal images of double immunostained sections showing that in the cortex of the newborn and adult rats, VEGF immunostaining is localized within NeuN-labeled neurons and GFAP-labeled astrocytes, respectively. In the hypothalamus of an adult control rat (**C**), intense VEGF immunostaining is detected within both neuronal cell bodies located in the core of the SON, and within astrocytic profiles located on the ventral border of the nucleus (arrows in **C**), whereas. Insets: merged confocal images of double immunostained sections showing that within the SON, VEGF immunostaining is associated with both OT- and VP-labeled neurons, and with GFAP-labeled astrocytes. Cx: cortex; GFAP: glial fibrillary acidic protein; NeuN: nuclear neuronal antigen; OT: oxytocin; VP: vasopressin; VEGF: vascular endothelial growth factor. Scale bars: A-B = 50 μm; C = 100 μm; insets = 25 μm.

Among a variety of identified angiogenic factors, VEGF is generally considered to play a major role in the processes of angiogenesis, maintenance of cerebral vasculature and permeability [25]. It has recently been reported that neuronal VEGF plays important role in the process of angiogenesis in the developing CNS [26]. In full agreement with these data, we observed that throughout extra-hypothalamic regions, VEGF-immunostaining was essentially associated with NeuN-labeled neurons in newborn rats of less that 25 days postnatal (dpn), (Fig. 6A), and with GFAP-labeled astrocytes in adult (2–3-month-old) rats (Fig. 6B). In the hypothalamus by contrast, intense to moderate VEGF-immunostaining was associated with magnocellular neurons labeled for VP or OT in both newborn and adult rats (Fig. 6C).

#### Osmotic stimulus increases the expression of VEGF within SON neurons

**Figure 7:**
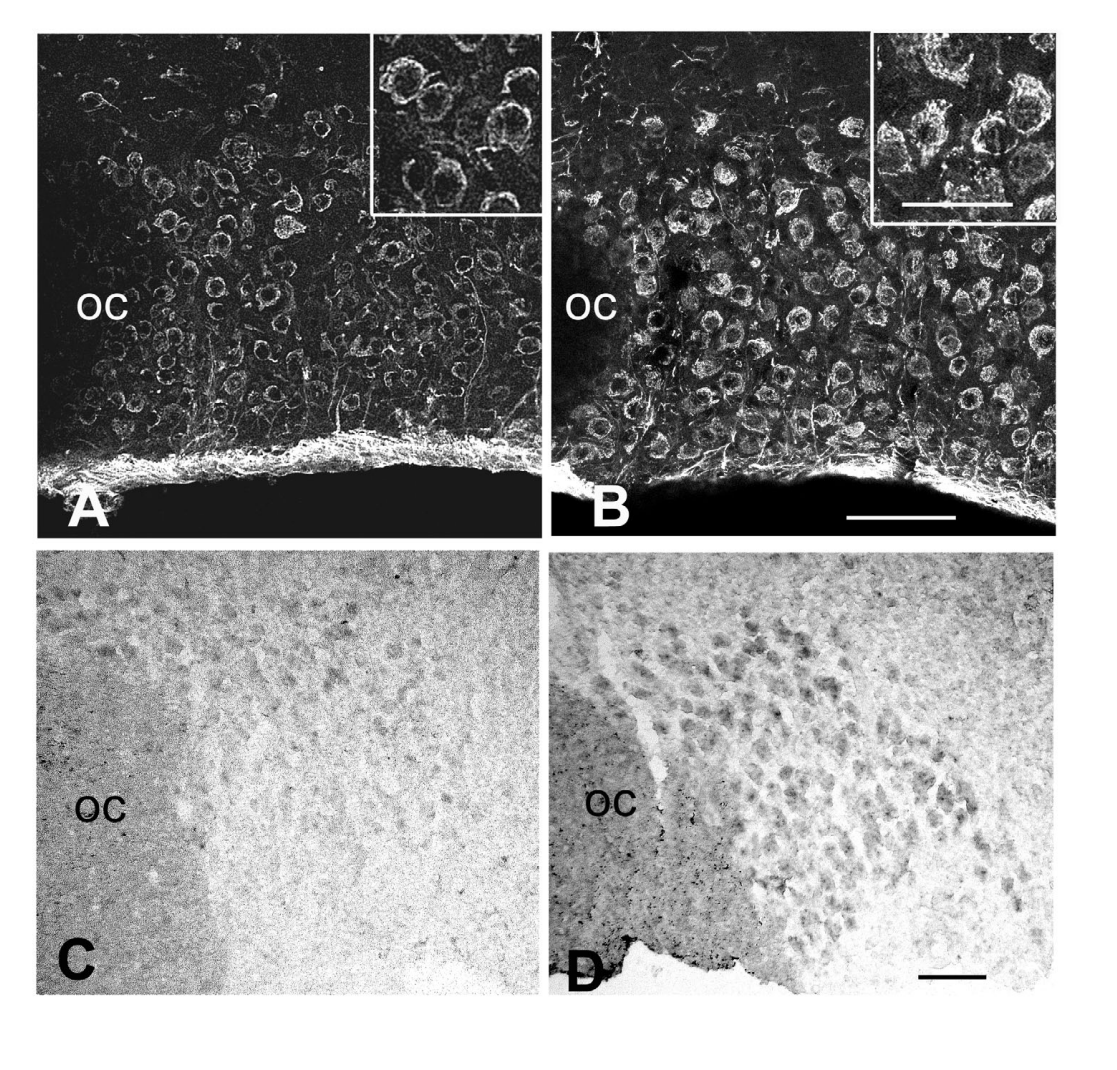
**Hyperosmotic stimulus increases VEGF expression within SON neurons**. **A-B**: Immunostaining for VEGF in control (**A**) and 6 days osmotically stimulated adult rats (**B**). Stack confocal images (10 μm-thick) of SON neurons show that moderate VEGF immunostaining is localized to perinuclear, golgi-like structures in the control rat (**A**), whereas intense immunostaining is dispersed throughout the cytoplasm in the stimulated rat (**B**) (insets show higher magnification of immunostained neurons). **C-D**: In situ hybridization for VEGF mRNA in control (**C**) and 6 days osmotically stimulated adult rats (**D**). Light microscope micrographs of 20 μm-thick cryostat sections showing that the mRNA labeling detected within the SON is highly increased in the stimulated (**D**) as compared with the control (**C**) rat. OC: optic chiasma; VEGF: vascular endothelial growth factor. Scale bars: A-B = 100 μm; C-D = 100 μm; insets = 50 μm.

In osmotically stimulated rats, VEGF immunostaining pattern was unchanged within astrocytes located within the SON or the surrounding brain regions whereas it was dramatically modified within SON neurons: whereas faint to moderate VEGF immunostaining was associated with perinuclear, golgi-like structures in SON neurons of control rats, intense VEGF immunostaining was detected throughout the neuronal cell bodies of stimulated rats (Fig. 7A–B).

In situ hybridization experiments for VEGF mRNA further indicated that the mRNA labeling detected within SON neurons was highly increased in 6 days stimulated rats as compared with control rats (Fig. 7C–D).

### Inhibition of endogenous VEGF impedes osmotically induced angiogenesis

#### Dexamethasone inhibits both neuronal VEGF expression and SON angiogenesis

**Figure 8:**
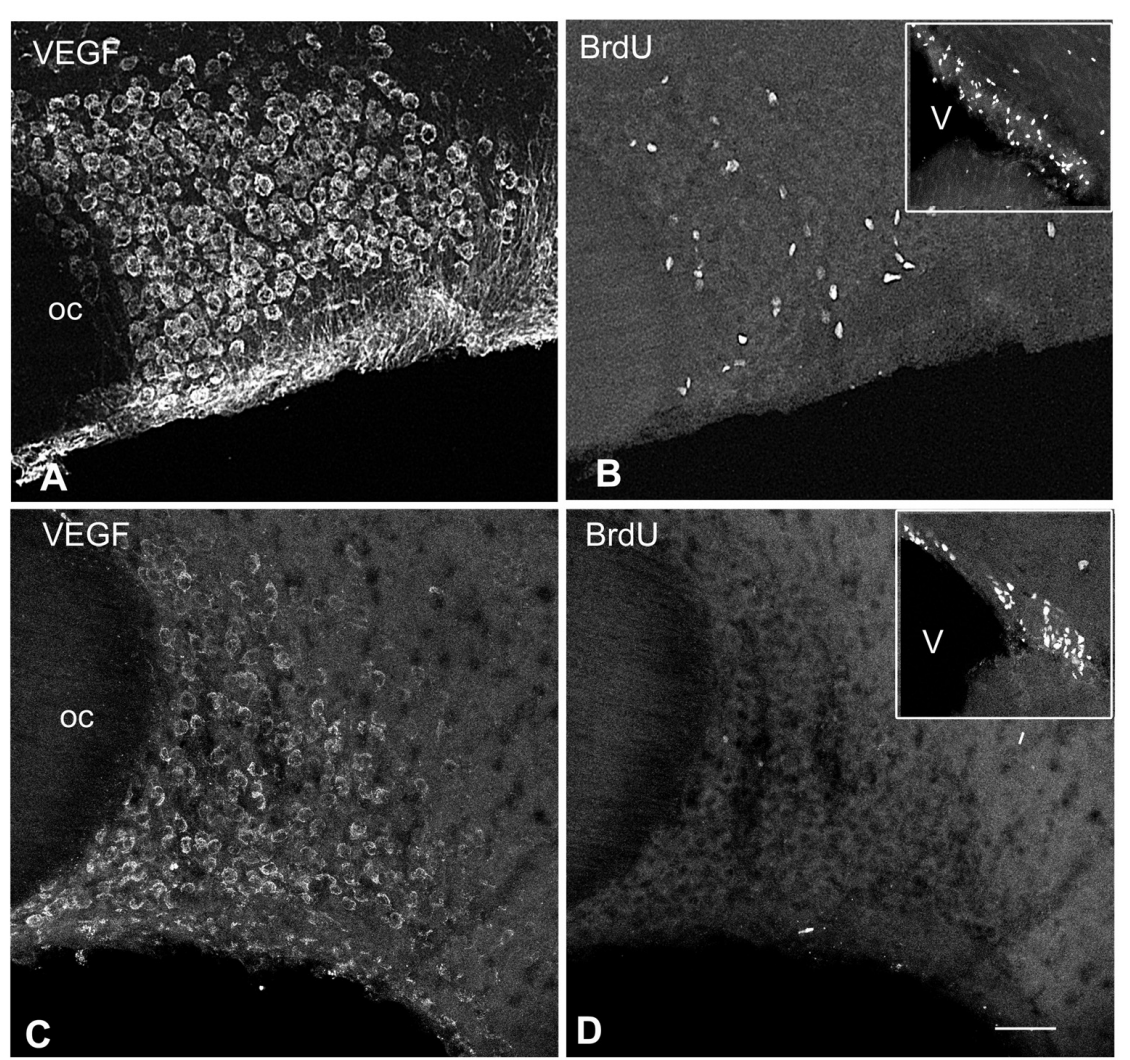
**Dexamethasone inhibits both VEGF expression and SON proliferative response**. Untreated (**A-B**) or Dexamethasone treated (**C-F**) rats were osmotically stimulated during 6 days and injected with BrdU 5 hours before their fixation. Stack confocal images (10 μm-thick) of sections double immunostained for VEGF and BrdU (**A-D**). As compared with the control rat (**A-B**), the SON of the dexamethasone treated rat exhibits decreased immunostaining for VEGF (**C**) and a complete disappearance of BrdU-labeled nuclei (**D**). By contrast, dexamethasone treatment has no effect on the BrdU-labeling of proliferative cells within the subventricular zone of the lateral ventricle (insets). Note that in the control rat, BrdU-labeled nuclei are essentially localized to the core of the SON that contains the VEGF-labeled neurons, and are scarce in the ventral portion of the nucleus that contains labeled astrocytes (stars in **A **and **B**). BrdU: bromodeoxyuridine; EBA: endothelial brain antigen; SON: supraoptic nucleus; VEGF: vascular endothelial growth factor. Scale bar: A-D = 100 μm.

Dexamethasone, a potent agonist of glucocorticoid receptors, has previously been shown to inhibit the expression of VEGF in various cell types [27-30]. In the present study, we thus evaluated the effects of chronic dexamethasone treatment of adult rats both on the expression of VEGF by magnocellular hypothalamic neurons and on the local angiogenic response induced by osmotic stimulus. Our data clearly show that in all dexamethasone-treated rats, VEGF immunostaining of magnocellular neurons was dramatically decreased as compared with control, untreated rats (Fig. 8A and 8C). In order to test whether dexamethasone treatment influenced the local proliferative response induced by osmotic stimulus, dexamethasone-treated adult rats were osmotically stimulated during 6 days, injected with BrdU, and sacrificed 5 hours later. Our data indicate that dexamethasone-treatment nearly abolished the angiogenic response to hyperosmotic stimulus within the SON and other magnocellular hypothalamic nuclei (Fig. 8B and 8D). On the other hand, dexamethasone treatment was found to only poorly modify the cell proliferation detected within the germinative zone of the lateral ventricle (Fig. 8B and 8D).

#### Local application of a blocking antibody to VEGF inhibits SON angiogenesis

**Figure 9:**
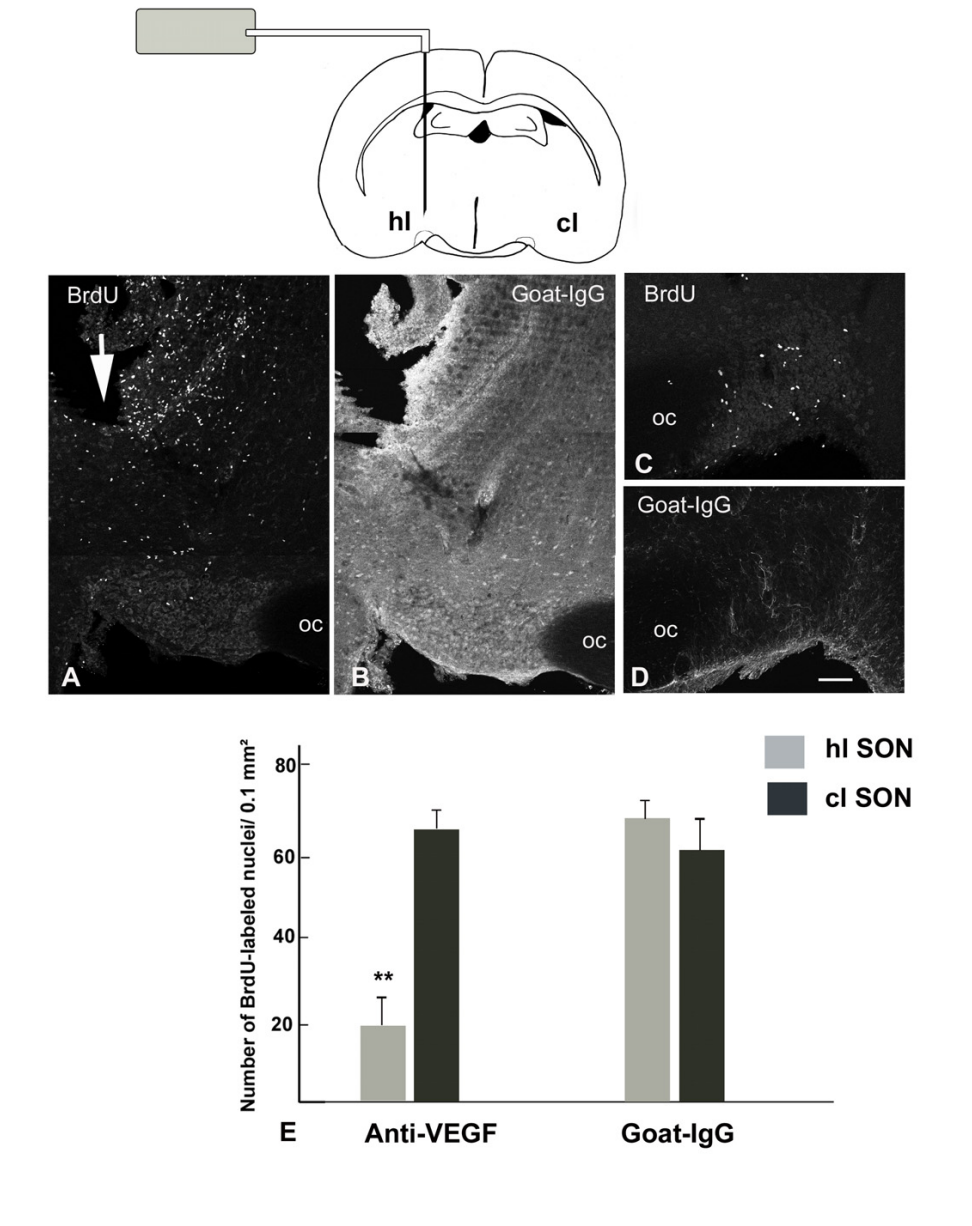
**VEGF antibody inhibits the SON proliferative response**. Rats were implanted unilaterally with a cannula positioned over the SON and connected to an Alzet osmotic pump containing a blocking VEGF antibody (schematic drawing), osmotically stimulated during 6 days, and injected with BrdU 5 hours before their fixation. **A-D**: Stack confocal images (10 μm-thick) of sections double immunostained for BrdU and goat-IgG. In the SON homolateral to the cannula, the number of BrdU-labeled nuclei is clearly lower that in the SON contralateral to the cannula (**A **vs **C**). Immunostaining for goat-IgG indicates that the goat-IgG anti-VEGF antibody has diffused from the cannula to the homolateral SON (**B**), but not to the contralateral SON (**D**). Note that numerous BrdU-labeled nuclei are observed along the border of the lesional cavity formed by the cannula diffusing the anti-VEGF antibody (arrow in **A**). **E**: Quantitative evaluation of the number of BrdU-labeled nuclei detected within the SON of 6 days osmotically stimulated rats implanted with a cannula diffusing either the anti-VEGF antibody or the isotype IgG. Data indicate that cell proliferation is significantly inhibited in the SON homolateral to implanted with cannulas diffusing blocking anti-VEGF antibody, but not with cannulas diffusing goat-IgG. Data represent means ± SEM of counts made on at least 4 SON areas per rats in 4 rats per experiment. Double asterisk: P < 0.01, Mann-Whitney test, statistically different from the SON contra-lateral to the cannula. BrdU: bromodeoxyuridine; cl: contra-lateral to the cannula; hl: homo-lateral to the cannula; IgG: type G immunoglobulin; OC: optic chiasma; SON: supraoptic nucleus; VEGF: vascular endothelial growth factor. Scale bar: A-D = 100 μm.

In order to inhibit endogenous VEGF, we then used a goat IgG VEGF neutralizing antibody. An intracerebral cannula connected to an osmotic minipump containing the blocking antibody or an IgG isotype was implanted in close vicinity to the SON of rats submitted to prolonged osmotic stimulus. In the implanted rats, the distribution of VEGF neutralizing antibody and of the IgG isotype was determined by the immunocytochemical detection of goat IgG: in all the implanted rats examined, infused IgG was found to diffuse throughout a 1 mm wide area surrounding the lesional cavity induced by the cannula, thus including the whole SON and part of the PVN homolateral to the cannula, whereas no diffusion could be detected within the magnocellular hypothalamic nuclei contralateral to the cannula (Fig. 9B and 9D). Our data indicated that in rats osmotically stimulated for 6 days, the cell proliferation (evaluated by the administration of BrdU, 5 hours prior to the fixation) was strongly inhibited in the SON adjacent to the cannula and, to a lesser extent, in the homolateral PVN, as compared with the magnocellular nuclei contra-lateral to the cannula (Fig. 9A, C and 9E). By contrast, a similar high rate of cell proliferation was detected in the hypothalamic magnocellular nuclei of both sides in control rats unilaterally infused with goat IgG (Fig. 9E). In all the rats infused with either the VEGF neutralizing antibody or the goat IgG, strong cell proliferation was detected throughout the area bordering the lesional cavity (Fig. 9A).

## Discussion

In mammals, the terminal CNS vascularization occurs postnatally via active angiogenesis, and thereafter this vasculature remains essentially quiescent under basal, non pathological conditions. In the present study we show that 1) the vascularization of hypothalamic magnocellular nuclei can be modified throughout adulthood via local angiogenesis induced by hyperosmotic stimuli and 2) such local angiogenic events are related to the expression of high levels of VEGF by magnocellular VP and OT neurons.

### Osmotic stimuli induce proliferation of SON capillary endothelial cells

More than 28 years ago, it has been established that prolonged stimulation of hypothalamic VP and OT neurons by hyperosmotic stimuli induced substantial proliferation of glial and endothelial cells within the supraoptic nucleus [11]. Although they fully confirm these previous data, our present data indicate that the cell proliferation occurring locally within hypothalamic magnocellular nuclei mostly involve endothelial cells, whereas proliferation of both endothelial cells and astrocytes was previously reported. A first explanation for such discrepant data is that the proliferation rate of astrocytes has been underestimated in the present study. Proliferative astrocytes were identified here as those cells immunostained for the astrocytic marker GFAP that exhibited a BrdU-labeled nucleus. Since newly formed astrocytes only express very low levels of GFAP[31,32], it is thus possible that proliferative astrocytes appeared GFAP-negative. In all the sections examined however, BrdU-labeled nuclei were preferentially located within the core of the SON, whereas the SON astrocytic cell bodies are localized to the ventral border of the nucleus. The differential evaluation of astrocytic proliferation may rather be related to marked differences in the methods used to label proliferative cells. In the present study, BrdU was injected 5 hours before the fixation of the rats (therefore labeling the cells that have undergone a process of cell division during this short period), while ^3^H-thymidine was previously injected twice daily during the whole duration of the hyperosmotic stimulation (14 days). It is thus very likely that under the present conditions, only those cells exhibiting high rate of proliferation were BrdU-labeled, whereas all the cells that have proliferated all along the period of osmotic stimulation were ^3^H-thymidine-labeled in the previous study. This clearly indicates that, in the SON of osmotically stimulated rats, the rate of proliferation of SON endothelial cells highly surpasses that of astrocytes.

We also show that within the SON of osmotically stimulated rats, high rate proliferation of capillary endothelial cells is accompanied by lower proliferation rate of NG2-labeled cells. Interestingly, the proteoglycan NG2 has been found to be expressed by vascular pericytes surrounding the nascent vessels [33-35]. Although the numerous NG2-labeled cells dispersed throughout the adult rat brain were initially identified as oligodendrocyte precusors [15], it is now generally admitted that these cells represent multipotent progenitor cells [16]. It can thus be assumed that at least part of these cells differentiate into vascular pericytes that participate in the formation and/or stabilization of new capillaries [36-38].

### SON angiogenesis reversibly modifies the local vasculature

The proliferation of endothelial cells, occurring either under physiological or pathological conditions, is generally associated with angiogenesis that leads to the formation of new capillary vessels. The idea that the proliferative response detected within the SON corresponds to local angiogenesis is strongly supported by the present data demonstrating that prolonged osmotic stimulation induced 1) pronounced modifications of the anatomical organization of SON capillary vessels, with both an expansion of a capillary network throughout the increased SON volume and an increase in the network density, and 2) an increased expression by SON capillaries of nestin and vimentin, two intermediate filament proteins highly expressed by newly formed endothelial cells [19,20,22,23]. Since prolonged osmotic stimuli induce strong activation of the functional activity of VP and OT magnocellular hypothalamic neurons [10], it can reasonably be assumed that the formation of new vessels within the SON will increase the circulating metabolic support to these neurons. A particularity of magnocellular neurons is that the size of their cell body increases during prolonged osmotic stimulation, leading to a progressive expansion of the volume occupied by the corresponding hypothalamic nuclei [11]. Newly formed vessels may thus also contribute to the vasularization of the expanded nucleus.

An intriguing question raised by the present findings concerns the fate of that SON new vessels formed during the period of osmotic stimulation. Our data clearly show that the density of the capillary network and the phenotypic expression of SON capillary vessels returned to control levels several days after the cessation of the osmotic stimulus. It is thus very likely that newly formed vessels are progressively deleted following rehydration. In control, normally hydrated rats, the SON vascularization however always remains particularly dense as compared with the surrounding regions, and the SON capillary vessels always express high levels of nestin. It can thus be assumed that, even under basal physiological conditions, new vessels are continuously added to the SON vasculature via sparse angiogenic events.

### Neuronal VEGF is the major stimulus for SON angiogenesis

The fact that osmotic stimulus induces reversible angiogenesis within the hypothalamic magnocellular nuclei strongly suggests that local secretion of potent angiogenic factor(s) occurs within these nuclei. Although a variety of angiogenic factors have been identified in the CNS, VEGF is generally recognized as the major factor involved in the processes of angiogenesis [25,39,40]. That endogenous VEGF plays a major role in the osmotically induced angiogenesis was suggested by the present findings that 1) contrasting with most CNS neurons, hypothalamic magnocellular neurons continue to express VEGF throughout adulthood, 2) this expression is highly increased by osmotic stimulation, and 3) osmotically induced angiogenic events are impaired when VEGF is inhibited by dexamethasone or by a blocking antibody.

Dexamethasone has been shown to inhibit VEGF expression in a large variety of tissue [27-30]. We show here that dexamethasone treatment of osmotically stimulated rats dramatically decreases the VEGF immunostaining associated with magnocellular neurons and, to a lesser extent, astrocytes. Our data further indicate that such decreased VEGF immunostaining of hypothalamic magnocellular neurons correlates with both a potent inhibition of cell proliferation within these nuclei, and a strong decrease of the nestin immunostaining of capillary vessels (not shown). This obviously supports the idea that endogenous VEGF is at least partly responsible for the local angiogenic events occurring within the hypothalamic magnocellular nuclei of hyperosmotically stimulated rats. Since hypothalamic magnocellular neurons of the adult rat express the glucocorticoid receptor [41], it is very likely that the dexamethasone effect observed here directly results from a direct repression of the VEGF gene. Glucocorticoids are however known to repress a variety of genes [42], which may also interfere with the angiogenesis described here. In order to demonstrate that endogenous VEGF is directly responsible for SON angiogenesis, we lastly performed a local infusion of a neutralizing antibody to VEGF that has been successfully used in previous studies to block endogenous VEGF activity in the rodent, both in peripheral organs [43,44]and within the brain [45]. An advantage of this antibody raised in the goat is that its diffusion throughout the brain parenchyma can easily be controlled. In the present study we show that the proliferative response to hyperosmotic stimulus was strongly inhibited within those hypothalamic nuclei that were located within this diffusion zone, but was not affected within the nuclei contra-lateral to the implanted cannula. Moreover, numerous proliferating cells were always detected along the border of lesional cavity induced by the cannula implantation, strongly suggesting that the blocking antibody specifically abolishes the VEGF-dependent cell proliferations, but has only poor effect on the proliferation of glial cells induced by CNS injury [46,47].

Although VEGF was also detected within SON astrocytes, our present observations suggest that the angiogenesis described here is preferentially related to the cytokine of neuronal origin. Within the SON of osmotically stimulated rats, BrdU-labeled proliferating cells were indeed always restricted to the core of the SON containing the VEGF-labeled neuronal cell bodies, and were scarce in the ventral portion of the nucleus containing the labeled astrocytes (Fig. 8 A–B). Moreover, osmotic stimulus was found to increase the expression of VEGF mRNA in the only SON neurons. In a series of previous studies, astrocytic VEGF has been associated with the angiogenesis occurring in various CNS pathological processes [48-50]. It could thus be assumed that, in the SON as in the other brain regions, neuronal VEGF constitutes the stimulus for physiological angiogenesis, whereas astrocytic VEGF is essentially involved in those angiogenic events occurring under pathological or traumatic conditions.

## Conclusion

In mammals, the final CNS vasculature is established during late postnatal development via active cerebral angiogenesis that is closely correlated with the neuronal expression of VEGF. Thereafter, neuronal VEGF is down-regulated parallely to an increased expression by glial cells, and under basal, non pathological conditions, the vasculature remains essentially quiescent [26]. In the present study we show that, a simple physiological stimulus can induce reversible angiogenesis within specific hypothalamic nuclei containing neurons that maintain VEGF expression throughout adulthood. Given the importance of angiogenic processes in a number of CNS diseases, these hypothalamic nuclei may thus provide a suitable model to elucidate the cellular and molecular mechanisms that control angiogenesis within the adult CNS.

## Methods

### Animals

Animals used were adult male Wistar rats of different ages, extending from 10 days to 3 months. They were housed in light (12 h dark and 12 h light) and temperature (21 ± 1°C) controlled rooms. Adult rats (2–3-month-old) were divided into three groups including 1) control rats that had free access to standard dry food and tap water, 2) hyperosmotically stimulated rats that were given 2% NaCl solution as drinking fluid during 1 to 10 days, and 3) rehydrated rats that were in hyperosmotically stimulated during 6 days and were then given tap water as drinking fluid during 1 to 5 days. All these animals were treated in accordance with the principles of laboratory animal care published by the French Ethical Committee.

### Implantation of intracerebral cannulas

After deep anesthesia with equithesine (3 ml/kg), animals were placed in a David Kopf stereotaxic device and a stainless-steel cannula (26 gauge, 10 mm long) was placed dorso-laterally to the right SON according to the stereotaxic atlas of Paxinos and Watson (1982): 1.3 mm posterior to bregma; 2.1 mm lateral; 8.5 mm dorsoventral (Fig. 9). The cannula was connected via a polyethylene tubing to a miniature Alzet osmotic pump (model 1007D, delivery rate 0.5 μl per hour during 7 days) filled up with either 1) a goat anti-human VEGF neutralizing antibody (R&D Systems, diluted 30 μg/ml in phosphate buffer saline, PBS) known to successively block rodent VEGF [51] (n = 5), or 2) normal goat IgG (30 μg/ml) (n = 4). The cannula was cemented in place adjacent to an anchor screw inserted in the skull, and the Alzet pump was inserted into a subcutaneous pocket made with sterile forceps over the rat neck and shoulder blades. Twenty four hours after the surgery, animals were given 2% saline as drinking fluid during 5 days and thereafter fixed by intracardiac perfusion after BrdU administration as described below.

### Dexamethasone treatment

Control rats (cont, n = 4) and dexamethasone-treated rats (dexa, n = 7) received a daily subcutaneous injection of 0.5 ml saline or dexamethasone (5 mg/kg in 0.5 ml saline), respectively, during 6 days. The first day of the treatment, they were given 2% saline as drinking fluid during 6 days and thereafter fixed by intracardiac perfusion after BrdU administration as described below.

### Administration of bromodeoxyuridine (BrdU)

BrdU (Sigma) was administered intraperitoneally (150 mg/kg in 0.5 ml of 0.01N hydroxy-chloride solution) 5 hours before the fixation of the animals.

### In situ hybridization

Adult rats either normally hydrated (control, n = 3) or drinking 2% saline during 6 days (osmotically stimulated, n = 3) were killed by decapitation. Brains were rapidly dissected, frozen at -40°C in isopentane and stored at -80°C. Coronal sections of hypothalamus (20 μm) were cut with a cryostat (Leica CM3000), thaw mounted on polylysin coated slides, fixed in 4% paraformaldehyde in PBS buffer (in mM: NaCl 130, Na_2_HPO_4 _7, NaH_2_PO_4 _3) for 5 min at 4°C, rinsed in PBS, dehydrated in increasing concentrations of ethanol (70 and 95%) and air dried. In situ hybridization was performed with oligonucleotide probes complementary to VEGF (482–529; Genbank accession n°AF215725) that did not match any known sequence in Genbank except those of the intended genes. Oligonucleotide probes were labeled at their 3' end with digoxigenin 11-dUTP using terminal transferase (Roche). Sections were incubated in a humid chamber overnight at 43°C with the primers (10 nM) in hybridization buffer. They were rinsed at 45°C in decreasing concentrations of saline-sodium citrate, followed by a final 30 min wash in the buffer supplemented with 5% BSA and 0.1% N-Lauroylsarcosine. Slides were then incubated 2–3 h with a sheep antidigoxigenin antibody coupled to alkaline phosphatase (Roche) diluted 1/500 in buffer supplemented with 5% BSA, carefully rinsed, and incubated 12 hours with nitroblue tetrazolium and 5-bromo 4-chloro 3-indolylphosphate (Roche). After mounting in Mowiol (Calbiochem, La Jolla, CA), slides were viewed under a light microscope. No hybridization signal was detected in control sections hybridized with a 50-mer random probe not complementary to any sequence in Genbank [52].

### Immunocytochemistry

After deep anesthesia with Equithesin, animals were either 1) decapitated and, after rapid dissection, the brain was fixed by immerssion in a solution of 4% paraformaldehyde 0.1 M phosphate buffer, pH 7, or 2) perfused through the ascending aorta with phosphate-buffered saline (PBS), pH 7.4, followed by 100 to 500 ml of fixative. The forebrain was dissected and fixed by immersion overnight in the same fixative. It was then cut frontally with a vibratome into 40–50-μm thick sections. These were carefully rinsed in PBS and subsequently treated for multiple fluorescence labeling.

The vibratome sections were incubated for 48 hours at 4°C with one or two primary antibodies including polyclonal rabbit IgG, polyclonal goat IgG, monoclonal mouse IgG or IgM, and monoclonal rat IgG antibodies (The different markers, vendors and concentrations used in this study are listed in Table 1). After rinsing in PBS, they were incubated for 4 hours at 4°C with corresponding secondary antibodies conjugated with Alexa-488 (Molecular Probes, Eugene, USA) or with Cy3 (Jackson Laboratories, USA). The primary and secondary antibodies were diluted in PBS containing 1% normal goat or donkey serum and 0.1% Triton X-100. Sections treated for the immunodetection of BrdU were incubated within 2N HCL for 30 minutes at room temperature, carefully rinsed in PBS, and incubated 48 h at 4°C with a mouse or rat monoclonal antibody anti-BrdU, either alone or in combination with another primary antibody.

**Table 1:** Antibodies used

Marker	Species	Working dilution	Vendor or productor
**Proliferation**			
BrdU	Mouse IgG	1:500	Novo Castra (Newcastle, UK)
BrdU	Rat IgG	1:500	Accurate Chemicals (New York, USA)
**Magnocellular neurons**			
OT	Rabbit IgG	1:2000	Produced by G. Alonso
VP	Rabbit IgG	1:2000	Produced by G. Alonso
**Astrocytes**			
GFAP	Rabbit IgG	1:5000	Dakko (Glostrup, Denmark)
**Microglia and endothelial cells**			
Lectin from bandeirae		1:2000	Sigma
Simplifolia (IsoB4)			
**Oligodendrocyte progenitors**			
NG2	Rabbit IgG	1:500	Chemicon (Temecula, USA)
**Vessels**			
Rat IgG-Alexa 488 conjugated	Goat IgG	1:2000	Molecular Probes (Eugene, USA)
EBA	Mouse IGM	1:5000	Sternberger Monoclonals (Lutherville, USA)
Nestin	Mouse IgG	1:100	Hybridoma Bank (Iowa city, USA)
Vimentin	Mouse IgG	1:5000	Sigma

BrdU: bromodeoxy uridine; EBA: endothelial brain antigen; GFAP: glial fibrillary acidic protein; IgG-IgM: immunoglobulins type G and M; OT: oxytocin; VP: vasopressin.

The specificity of the vasopressin, oxytocin and of commercial antibodies has been assessed by absorption tests. Additional controls consisted of (1) omitting the primary antibodies and applying the secondary antibodies alone, (2) applying each primary antibody sequentially and then reacting them with an inappropriate secondary antibody, and (3) exciting each fluorochrome by the inappropriate illumination. This allowed us to confirm that the two secondary antibodies used in double immunostaining experiments did not induce artifactual fluorescent labelling and that there was no overlap of the emission spectra of the two fluorochromes.

### Imaging and quantification

After rinsing in PBS, labeled sections were mounted in Mowiol and observed under a Biorad MRC 1024 confocal laser scanning microscope equipped with a krypton/argon mixed gas laser. Two laser lines emitting at 488 nm and 568 nm were used for exciting GFP and the Alexa-488 or Cy3-conjugated secondary markers. The background noise of each confocal image was reduced by averaging four image inputs. The organization of the immunostained structures was studied 1) on single confocal images or 2) on bi-dimensional reconstructed images obtained by collecting 5 to 10 consecutive confocal images 1 μm apart through the whole vibratome section thickness, and by projecting on the same plane. Unaltered digitalized images were transferred to a PC type computer and Adobe Photoshop was used to prepare final figures.

Quantitative analysis was performed on series of sections passing through the middle portions of the SON (i.e. the largest SON areas): 1) The cell proliferation was quantified by counting the BrdU-labeled nuclei detected in four SON areas per rat, in at least three rats per experiment, 2) The nature of proliferative cells was evaluated on sections double-labeled for BrdU and a specific cell type marker. The number of double-labeled cells was pooled among at least 4 SON areas per rat, in 4 rats, and 3) The anatomical organization of SON vessels was evaluated on sections of rat brains fixed by immerssion and double immunostained for Rat-IgG and for VP + OT. The density of SON vessels was quantified according to the point-counting method [53], by scoring the number of point intersections of Rat-IgG-labeled vessels with a grid centered either on the SON area containing the neurons labeled for VP or OT or on the lateral hypothalamus overlying the SON. The total amount of SON vessels was evaluated by measuring the surface occupied by Rat-IgG-labeled vessels (% of fluorescent pixels on binary images) within the SON area containing the VP + OT-labeled cell bodies (with the Image Tool image analysis system, University of Texas Health Science Center, San Antonio, USA). All quantifications were performed on at least 4 SON areas per rat, and for at least 3 rats per experiment. Data were statistically compared by using the non parametric test of Mann and Whitney.

## List of abbreviations used

AN: accessory magnocellular nuclei

BrdU: bromodeoxyuridine

CNS: central nervous system

EBA: endothelial brain antigen

GFAP: glial fibrillary protein

IgG: immunoglobulin type G

IsoB4: isolectin B4

NeuN: neuronal nuclear antigen

NG2: NG2 chondroitin sulfate proteoglycan

OC: optic chiasma

OT: oxytocin

PVN: paraventricular nucleus

SON: supraoptic nucleus

VP: vasopressin

VEGF: vascular endothelial growth factor

## Authors' contributions

GA conceived the study, conducted most experiments and wrote the paper; EG and AG conducted the immunocytochemical experiments; AV conducted the in situ hybridization experiments.

**Table 2:** Quantitative evaluation of the nature of SON proliferative cells

		% of BrdU-labeled nuclei associated with specific cell markers
**Cell type**	**Marker**	

Neurons	VP + OT	0
Astrocytes	GFAP	6
Oligo. Precursors	NG2	18
Microglia	IsoB4	8
Vessels	IsoB4	60
Vessels	Nestin	67
Vessels	EBA	55

BrdU was administrated to osmotically stimulated rats drinking 2% saline during 6 days, and the animals were fixed 5 hours after the BrdU administration. The relative proportion of each BrdU-labeled cell type was calculated from at least 4 sections per rat in 4 stimulated rats. More than 500 BrdU-labeled nuclei were scored for most markers.

GFAP: glial fibrillary acidic protein; EBA: endothelial brain antigen; NG2: proteoglycan NG2; OT: oxytocin; VP: vasopressin.
